# Ancient homeobox gene loss and the evolution of chordate brain and pharynx development: deductions from amphioxus gene expression

**DOI:** 10.1098/rspb.2010.0647

**Published:** 2010-06-16

**Authors:** Thomas Butts, Peter W. H. Holland, David E. K. Ferrier

**Affiliations:** 1 Department of Zoology, University of Oxford, South Parks Road, Oxford OX1 3PS, UK; 2 Scottish Oceans Institute, University of St Andrews, East Sands, St Andrews, Fife KY16 8LB, UK

**Keywords:** cephalochordate, homeobox, gene loss, pharynx evolution, brain evolution

## Abstract

Homeobox genes encode a large superclass of transcription factors with widespread roles in animal development. Within chordates there are over 100 homeobox genes in the invertebrate cephalochordate amphioxus and over 200 in humans. Set against this general trend of increasing gene number in vertebrate evolution, some ancient homeobox genes that were present in the last common ancestor of chordates have been lost from vertebrates. Here, we describe the embryonic expression of four amphioxus descendants of these genes—*AmphiNedxa, AmphiNedxb, AmphiMsxlx* and *AmphiNKx7*. All four genes are expressed with a striking asymmetry about the left–right axis in the pharyngeal region of neurula embryos, mirroring the pronounced asymmetry of amphioxus embryogenesis. *AmphiMsxlx* and *AmphiNKx7* are also transiently expressed in an anterior neural tube region destined to become the cerebral vesicle. These findings suggest significant rewiring of developmental gene regulatory networks occurred during chordate evolution, coincident with homeobox gene loss. We propose that loss of otherwise widely conserved genes is possible when these genes function in a confined role in development that is subsequently lost or significantly modified during evolution. In the case of these homeobox genes, we propose that this has occurred in relation to the evolution of the chordate pharynx and brain.

## 1. Introduction

Homeobox genes encode a large group of transcription factors that expanded very early in metazoan evolution (Ryan *et al*. 2006), and the diversification of these genes has been implicated in the evolution of the diversity of animal body plans. Frequently, homeobox genes have major directive roles in developmental gene-regulatory networks and many, such as the *Hox* genes in the context of the anterior–posterior axis (Deschamps 2007; Duboule 2007) and *Pax6* in the case of eye specification (Gehring 2002), function in aspects of development that are highly conserved across bilaterian taxa. Our understanding of the evolution of the homeobox genes, and of animal development more generally, has in recent years been greatly impacted by the progressive advance of genome-sequencing projects across the Metazoa. In this regard, the genome of the chordate amphioxus (*Branchiostoma floridae*; Putnam *et al*. 2008) has been particularly revealing.

The lancelets, or amphioxus, were long thought to be the closest invertebrate relatives of the vertebrates, but recent molecular evidence has led to their relocation to the most basal lineage within the chordates (Bourlat *et al*. 2006; Delsuc *et al*. 2006; Putnam *et al*. 2008). Notwithstanding this repositioning, amphioxus remains a most informative extant outgroup taxon for studying the early evolution of the vertebrates (Holland & Chen 2001). In large part, this importance is a result of the derived nature of development in the other invertebrate chordate lineage, the urochordates. In contrast, amphioxus in many ways resembles a typical vertebrate: gastrulation is coordinated by an organizer (Yu *et al*. 2007), and is followed by neurulation, producing an animal with a subepidermal dorsal hollow nerve cord (Holland & Holland 1999) that is enlarged, albeit not very much, at its anterior end, where the various sense organs are situated, and that is surrounded laterally and ventrally by mesoderm that develops from somites (Beaster-Jones *et al*. 2008). A notochord sits atop a through gut that at the anterior end includes a muscular pharynx. Following a larval stage, amphioxus undergoes a metamorphic transition that is homologous to that seen in amphibians (Paris *et al*. 2008*a*,*b*), and as an adult even resembles a small, poorly cephalized fish.

Classical descriptive morphological studies (Hatschek 1893; Conklin 1932) underlined the differences as well as the similarities between the lancelets and their vertebrate cousins, and the embryological and larval development of amphioxus differs from that of vertebrates in a number of important ways. Amphioxus possesses unpaired sense organs: a single photoreceptive eyespot (though this is not the only photoreceptive structure), and a single putative balance organ located in the cerebral vesicle (Lacalli & Kelly 2000) but no clear olfactory organ (Lacalli 2004). The notochord of amphioxus, unlike its vertebrate counterparts, is muscular (Flood *et al*. 1969) and extends the full length of the body, acting as an antagonist to the lateral muscles during swimming or burrowing (Guthrie & Banks 1970). The formation of the mesodermal somites also differs between amphioxus and vertebrates, with the first eight somites in *B. floridae* (Holland *et al*. 1997) forming by enterocoely rather than schizocoely, which instead occurs during the addition of more posterior somites in amphioxus and in vertebrate development. Interestingly, the somites are generally asymmetric with the right series offset by half a somite length to the posterior relative to the left series, a result of their alternating production from the posterior growth zone (Schubert *et al*. 2001). Perhaps the most striking difference is the asymmetry that accompanies the development of the pharyngeal region of the amphioxus neurula. The mouth opens on the left-hand side, just behind a pre-oral pit, a product of the fusion of the left anterior head cavity with the ectoderm. Both these structures have no equivalent on the right-hand side, where the enigmatic club-shaped gland develops just below the endostyle, the presumed thyroid homologue (Ogasawara 2000). Posterior to these, the first few gill slits form ventrally and migrate to the right side, where they break through the body wall (Whittaker 1997). Thus, despite the extensive similarity and homology, amphioxus embryogenesis and larval development differs from that of vertebrates in several important aspects, not least of which is profound asymmetry in early development.

In genomic terms, amphioxus has retained an unprecedented number of ancestral features, both in terms of gene organization at the microsyntenic (e.g. Ferrier *et al*. 2005; Mazet *et al*. 2006; Butts *et al*. 2008; Horton *et al*. 2008) and macrosyntenic (Castro & Holland 2003; Putnam *et al*. 2008) scales, and in terms of gene retention. Indeed, following the completion of the genome project, several genomic surveys of different gene families have underscored the conclusion that amphioxus generally has undergone comparatively little gene loss in its evolutionary history (D'Aniello *et al*. 2008; Holland *et al*. 2008; Paris *et al*. 2008*b*; Schubert *et al*. 2008; Shimeld 2008; Yu *et al*. 2008; Dai *et al*. 2009).

Interestingly, in regard to the homeobox gene complement, amphioxus has retained members of seven gene families (Abox, Bari, Msxlx, Nedx, NK7, Repo and Rough) that date back to at least the last common ancestor of protostomes and deuterostomes, but have been lost from the vertebrate lineage (Holland *et al*. 2008; Takatori *et al*. 2008). The early evolution of vertebrates was characterized by whole-genome duplications (Dehal & Boore 2005; Putnam *et al*. 2008), and the recruitment of duplicated genes and gene-regulatory networks to developmental innovations is one of the key themes of vertebrate developmental evolution. Given that homeobox genes frequently function in developmental cascades in determinitive roles, specifying embryonic territories and fates, the loss of ancient homeobox genes presents a particularly interesting contrast to the genetic expansion widely described for early vertebrate evolution. In order to shed light on this transition, we have examined the developmental expression of the amphioxus orthologues of the pan-bilaterian homeobox gene families lost from vertebrates. We find that four genes are detectably expressed during amphioxus embryogenesis, and that intriguingly, all these four are expressed in territories that, in vertebrates, have undergone significant modification since the last common ancestor of the chordates: the anterior central nervous system and the pharynx.

## 2. Material and methods

### (a) Animal collection

Adult amphioxus, *Branchiostoma floridae*, were collected in July and August 2006 from Old Tampa Bay, FL, USA and fertilized *in vitro* as described by Holland & Holland (1993). Developing embryos were fixed for *in situ* hybridization at regular intervals by incubation for 60 min at room temperature or overnight at 4°C in 4 per cent PFA in MOPS buffer (0.1 M MOPS, 0.5 M NaCl, pH 7.0). After fixation, embryos were washed twice in 70 per cent ethanol and stored at −20°C in 70 per cent ethanol.

### (b) Probes

All aqueous solutions for subsequent stages were treated for 2 h with 0.5 per cent diethyl pyrocarbonate (DEPC) and autoclaved as a precaution against RNAse contamination. Probes were obtained by polymerase chain reaction on amphioxus genomic DNA obtained by phenol–chloroform extraction from fixed adult specimens. Primers to exonic sequences were designed using the v. 1.0 genome assembly of *Branchiostoma floridae* (http://genomeportal.jgi-psf.org/Brafl1/Brafl1.home.html). Primers used to clone exons of *AmphiNedxa*, *AmphiNedxb*, *AmphiMsxlx* and *AmphiNKx7* were as follows: BfNedxaex1f, 5′-ATGTCGGGGTCTGATAACC-3′, BfNedxaex1r, 5′-CTTCTGTTCGCAAGTTGTTGA-3′; BfNedxbex1f, 5′-ACGTGCAGGAGAGGGAGAG-3′; BfNedxbex1r, 5′-TCGCTTTCATCTTCTTGCTG-3′; BfMsxlxex1f, 5′-GGCACTCCTATCCCACTTGT-3′; BfMsxlxex1r, 5′-GAGTTTTGGCGGTTTGTACC-3′; BfMsxlxex2f, 5′-ACGAAAAGATGGGAGCAAGA-3′; BfMsxlxex2r, 5′-GATTTTCGGACAGGTTGAGC-3′; BfMsxlxex3f, 5′- CGAGCCCGAGAGAGACGA-3′; BfMsxlxex3r, 5′- TCAATAGGTGAACGACACAGGAG-3′; BfNk7ex1f, 5′-GGCGATGCAGCAGGAGTC-3′; BfNk7ex1r, 5′- CTCGGAGTCAGAGTCTTCTCGC-3′; BfNk7ex3f, 5′-TCTGGTTCCAAAATCGGCG-3′; BfNk7ex3r, 5′-TAGTCCGTGTGGCACGTTTG3′. The probe for *AmphiNedxa* consisted of 373 bp in exon 1, for *AmphiNedxb* 363 bp of exon 1, for *AmphiMsxlx* an equimolar mixture of probes of length 514, 161 and 210 bp from exons 1, 2 and 3, respectively, and for *AmphiNKx7* an equimolar mixture of probes of length 298 and 353 bp from exons 1 and 3, respectively.

Gene fragments were cloned into pGEMT-easy (Promega). Plasmid DNA was isolated using QIAprep Spin Mini Kit (Qiagen) according to the manufacturer's instructions. Fragments were re-amplified from minipreps using M13 primers. This re-amplification product was run on a gel, excised and purified using the GFX gel extraction kit (Amersham).

Antisense and sense (control) probes were transcribed *in vitro* using DIG-RNA labelling (Roche) according to the manufacturer's instructions. The reaction was incubated at 37°C for at least 2 h. One microlitre of probe was run on an agarose gel to check for complete transcription and the probes were subsequently cleaned using Mini Quick Spin RNA columns (Roche) and then precipitated with 70 per cent ethanol and 100 mM LiCl. The probes were then washed with 70 per cent ethanol, dried, resuspended in DEPC-treated H_2_O and stored at −20°C.

### (c) *In situ* hybridization

*In situ* hybridizations were performed essentially as described elsewhere (Holland *et al*. 1996; Osborne *et al*. 2009) with the following modifications. After rehydration, embryos were digested with 7.5 µg ml^−1^ Proteinase K for 5–30 min depending on the size and stage of development. Mid-neurula stages (9/10 somites; 15 h development) were digested for 7 min. Late neurula stages (12 somites; 18 h development) were digested for 10 min. Prehybridization was conducted at 50–65°C with gentle shaking for at least 3 h. Hybridization was undertaken overnight at the same temperature with 50–200 ng of labelled probe. The embryos were blocked in 10 per cent sheep serum (in phosphate-buffered saline–Tween buffer) for at least 3 h at room temperature and then incubated in preabsorbed 1 : 1500 anti-DIG-alkaline phosphatase (Roche) at 4°C overnight.

Following staining, embryos mounted in 80 or 100 per cent glycerol were visualized and photographed under a Zeiss Axioskop 2 microscope. All digital images were processed with Adobe Photoshop, with adjustments to brightness, colour balance and contrast being made uniformly across the entirety of each image.

## 3. Results

**Figure 1.**
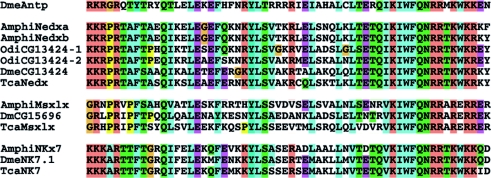
Homeodomain alignment of ancient gene families lost in vertebrates. The gene families Nedx, Msxlx and NK7 are aligned to the *Drosophila Antennapedia* homeodomain. As with other homeobox gene families, diagnostic family-specific residues are found throughout the homeodomain. Species abbreviations are as follows: Dme, *Drosophila melanogaster*; Amphi, *Branchiostoma floridae*; Tca, *Tribolium castaneum* and Odi, *Oikopleura dioica*.

The ancient homeobox genes that are the subject of this study have been identified previously and classified based upon phylogenetic reconstruction (Holland *et al*. 2008; Takatori *et al*. 2008). As with most ancient homeobox gene families, phylogenetic classification at the family level is robust and reflects the conservation of diagnostic residue combinations within the homeodomain (figure 1). The four genes studied here belong to three families: Nedx, Msxlx and NK7, and are all expressed in specific spatio-temporal patterns during amphioxus embryogenesis, only within the first day of amphioxus development.

### (a) *AmphiNedx* expression

**Figure 2.**
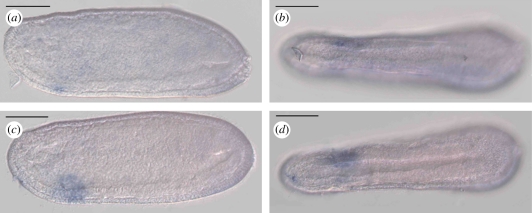
*Nedx* expression. (*a*,*b*) *AmphiNedxa* and (*c*,*d*) *AmphiNedxb* are both expressed at the mid-neurula stage in ventrolateral pharyngeal epidermis on the left-hand side, though the expression of *AmphiNedxb* is considerably stronger and covers a much broader area. (*a*,*c*) Lateral views, (*b*,*d*) ventral views. Anterior is to the left in all panels; scale bar, 50 µm.

The amphioxus genome possesses two lineage-specific duplicates of Nedx: *AmphiNedxa* and *AmphiNedxb* (Takatori *et al*. 2008). We find that both are expressed in an overlapping pattern solely in the neurula stage of amphioxus embryogenesis, although *AmphiNedxb* is expressed in a broader territory and at higher levels than *AmphiNedxa*. Expression is observed in the 9-somite neurula in a region of ventrolateral epidermis on the left-hand side of the anterior portion of the embryo (figure 2). At the 12-somite stage, approximately 3 h later, expression is no longer detectable (data not shown). At this stage in embryogenesis, many of the morphological features of the ventral pharyngeal region are yet to develop and the transient nature of the expression coupled with the lack of lineage tracing studies in amphioxus makes it hard to identify whether Nedx expression is confined to a particular organ-specific territory. In this regard, comparison with the previously reported expression patterns of *AmphiPax3/7* (Holland *et al*. 1999), *AmphiPax2/5/8* (Kozmik *et al*. 1999) and *AmphiSix4/5* (Kozmik *et al*. 2007) is informative. *AmphiPax2/5/8* is expressed in a number of tissues including asymmetrical structures. The strongest pharyngeal expression at neurula stages is endodermal and is observed in a region that at the early larval stage will mark the position of the mouth. In the early larva, as the mouth breaks through the body wall, the expression marks both the ectoderm and endoderm surrounding the mouth, which are in the process of fusing (Kozmik *et al*. 1999). In contrast, the expression of *AmphiPax3/7* in the neurula stage is much broader throughout the mesendoderm in the anterior third of the embryo. In the mouth region of the early larva, it specifically marks the endoderm both dorsal and ventral of the mouth opening (Holland *et al*. 1999). *AmphiSix4/5* is also widely expressed in the developing pharyngeal endoderm at neurula stages, but in and around the opening larval mouth its expression is confined to the endoderm located ventral to the mouth opening (Kozmik *et al*. 2007). The expression of the Nedx paralogues is comparable with the ventral half of the pharyngeal *AmphiPax2/5/8* territory in the neurula stage, though it is epidermal. Potentially these ectodermal cells then proceed to locate to the region ventral of the mouth opening in a position adjacent to the *AmphiSix4/5*-expressing cells, though this is speculative as by the larval stages of development Nedx expression is not detectable by *in situ* hybridization.

### (b) *AmphiMsxlx* expression

**Figure 3.**
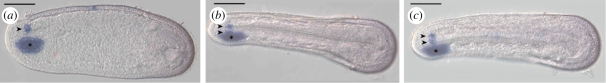
*AmphiMsxlx* expression. *AmphiMsxlx* is expressed at the mid-neurula stage in the left anterior gut diverticulum (asterisks) and in bilateral spots in the ventral anterior neural plate (arrowheads). (*a*) Lateral view; (*b*) dorsal view; (*c*) is rotated slightly laterally, off dorsal, to reveal these bilateral spots more clearly. Anterior is to the left in all panels; scale bar, 50 µm.

*AmphiMsxlx* is expressed in two territories during amphioxus embryogenesis: the anterior central nervous system and the left anterior gut diverticulum of the nine-somite neurula (figure 3). The neural expression is in the ventral half of the neural tube and is situated at the same anteroposterior level as the neuropore, within the territory that will develop into the cerebral vesicle. Expression does not extend to the anterior tip of the cerebral vesicle but is within the anterior vesicle, which is putatively homologous to the vertebrate diencephalon (Lacalli 2008). A second site of *AmphiMsxlx* expression is in the anterior endoderm. *AmphiMsxlx* is expressed here solely on the left-hand side in Hatschek's left diverticulum, which will go on to fuse with the ectoderm at the left-hand body wall and form the larval pre-oral pit, the amphioxus homologue of the vertebrate adenohypophysis (Candiani *et al*. 2008).

### (c) *AmphiNKx7* expression

**Figure 4.**
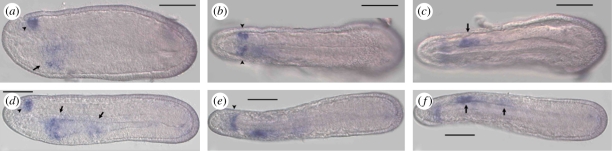
*AmphiNKx7* expression. *AmphiNKx7* is expressed at the (*a*–*c*) mid- (nine somites) and (*d*–*f*) late (12 somites) neurula stages in the anterior neural plate (arrowheads) and in the left side of the pharyngeal endoderm (arrows). (*a*,*d*) Lateral views; (*b*,*e*) dorsal views; (*c*,*f*) ventral views. Anterior is to the left in all panels; scale bar = 50 µm.

*AmphiNKx7*, the amphioxus orthologue of the *NK7.1* gene of *Drosophila*, is also expressed in a left-sided territory in the anterior endoderm and an anterior neural territory in the region that will develop into the cerebral vesicle (figure 4) in the neurula stage. The expression of *AmphiNKx7* is slightly less transient than the other genes discussed here and its expression is detectable at very low levels in the neural ectoderm just after hatching (roughly 12 h post-fertilization) and persists in the 12-somite stage (figure 4), though is not detectable at the time of mouth opening at 24 h post-fertilization (data not shown). The endodermal expression of *AmphiNKx7* is qualitatively weaker than the neural expression and diffusely covers a relatively extended area, especially at the 12-somite stage (figure 4).

## 4. Discussion

### (a) Gene loss and the rewiring of gene-regulatory networks

The expression of *AmphiNedxa*, *AmphiNedxb*, *AmphiMsxlx* and *AmphiNKx7* has important implications for the evolution of the structures in which they are expressed: the anterior central nervous system and the pharynx. The expression of *AmphiMsxlx* in Hatschek's left anterior diverticulum, and its inferred role in the early development of the pre-oral pit along with *POU1F1/Pit-1*, is an instance where expression resides within a structure that has vertebrate homologues, in this case the adenohypophysis (Candiani *et al*. 2008). A scenario can be envisaged whereby amphioxus represents the ancestral condition and the loss of *Msxlx* expression from this territory in vertebrates was associated with a change in the function of the ancestral adenohypophysis-like organ from external secretion to internal secretion of peptide hormones. An alternative possibility is that the function of *Msxlx* in the homologue of the adenohypophysis is a derived feature of amphioxus embryogenesis. Whether *Msxlx* exists and performs a similar function in hemichordates, perhaps in the protocoel and proboscis pore, will be of considerable importance in choosing between these hypotheses and in clarifying the evolution of the coelomopore complex and an adenohypophysis-like organ.

A whole suite of genes have been found to be expressed in the pre-oral pit including *AmphiEomes/Tbr1* (Satoh *et al*. 2002), *AmphihairyA*, *AmphihairyD* (Minguillón *et al*. 2003), *AmphiVent* (Kozmik *et al*. 2001), *AmphiPOU1F1/Pit-1* (Candiani *et al*. 2008), *Amphilhx3* (Wang *et al*. 2002) and *AmphiPax-6* (Glardon *et al*. 1998). Importantly, the expression of *AmphiPOU1F1/Pit-1*, which is one of the earliest specific markers of the vertebrate adenophypophysis and amphioxus pre-oral pit (Candiani *et al*. 2008) is first detected at the same stage as the *AmphiMsxlx* expression reported here, implying that *AmphiMsxlx* is one of the earliest genes active in the initial phase of the development of this organ. Two of the genes mentioned above, namely *Amphilhx3* and *AmphiPax-6* are expressed in the same territories as *AmphiMsxlx* in both the neuroectoderm and the endoderm. Thus, *AmphiMsxlx* may act as part of a regulatory circuit of transcription factors in concert with these genes during the development of both the pre-oral pit and the brain of amphioxus.

In common with the *AmphiMsxlx* gene, *AmphiNKx7* and the two *Nedx* paralogues—*AmphiNedxa* and *AmphiNedxb*—are expressed asymmetrically on the left-hand side during early pharyngeal development. However, the asymmetrical expression of the latter three genes is more caudal than that of *AmphiMsxlx*, in the regions where the mouth and first gill slit will later break through the body wall. The endodermal expression of *AmphiNKx7* is at the same anteroposterior position within the embryo as the ectodermal *Nedx* expression and accords well with the pharyngeal expression of *AmphiPax2/5/8* (Kozmik *et al*. 1999). In addition, the pattern is almost identical to that of *AmphiNK2-2* in this region, though this gene is expressed in the mid- and hindgut also (Holland *et al*. 1998). It is tempting to speculate therefore that the function of *AmphiNKx7*, the *AmphiNedx* genes, *AmphiPax2/5/8* and *AmphiNK2-2* are intertwined in specifying aspects of the asymmetric early development of pharyngeal structures in the amphioxus embryo, and that *NKx7* and *Nedx* have been lost in the vertebrate and ascidian lineages because of modulations in the developmental programme controlling pharyngeal development.

The expression of Nedx has also been examined in *Drosophila* (Nedx is *CG13424* in *D. melanogaster*) where it is specific to developing larval muscle, initially in the thoracic segments but later in all segments (Tomancak *et al*. 2007). It has recently been found to be a direct target of Twist, the central transcription factor in the early development of *Drosophila* mesoderm (Sandmann *et al*. 2007). The amphioxus *Twist* gene is likewise expressed during early mesodermal differentiation (Yasui *et al*. 1998) and its expression does not overlap with Nedx, implying that Nedx possesses distinct, non-homologous developmental roles in *Drosophila* and amphioxus.

The implication of changes in transcription factor networks is mirrored in the brain. The anterior end of the neural tube in amphioxus has been homologized with the vertebrate anterior central nervous system based upon both neuroanatomical and developmental genetic studies (Williams & Holland 1996; Lacalli 2004; Holland 2005). In both taxa, the anterior border of the Hox gene-expressing hindbrain is caudal to a domain expressing Otx family genes (Williams & Holland 1996). The expression of *AmphiMsxlx* and *AmphiNKx7* within the *AmphiOtx*-positive region of the cerebral vesicle suggests that these genes act to regionalize this structure in a manner that is accomplished in a different way in its putative vertebrate homologue, the diencephalon. The expression of *AmphiNKx7* is located posterior to that of *AmphiMsxlx* and possibly the two genes act to regionalize the *AmphiOtx*-expressing cerebral vesicle. This makes the loss of these genes in vertebrates particularly striking as it implies profound regulatory change during the evolution of a well-conserved part of the ancestral chordate brain, the diencephalon (Williams & Holland 1996; Holland & Holland 1999).

### (b) The gain (or loss) of asymmetry

The profound asymmetry of the amphioxus embryo has to date been largely ignored in molecular terms owing to the understandable concentration upon those many features that are conserved between amphioxus and other bilaterians, particularly the vertebrates. Traditional embryological study has led to the hypothesis that the mouth and the club-shaped gland represent modified gill slits (Whittaker 1997), a theory that is supported by the presence of Hatschek's nephron above the mouth, coupled with the actual gill slit pairs each being accompanied by a pair of nephrons, and the fact that the club-shaped gland has no known homologue in other chordate phyla. The corollary is that the original primordial mouth has been lost, with the implication that the asymmetric nature of amphioxus is secondarily derived.

Data from developmental genetic studies over the past decade have further refined the question of the origin of amphioxus pharyngeal asymmetry with many genes possessing sites of expression on one side of the pharyngeal endoderm in addition to sites in other developmental contexts that are conserved with vertebrates. Crucially though, the ectodermal invagination that will form the mouth, the stomodeum, has been homologized across chordates (Christiaen *et al*. 2007) using the expression of *Pitx* downstream of conserved ventralizing BMP signalling (Yu *et al*. 2007). This pattern of mouth development, and a loss of *Brachyury* and *Goosecoid* expression, is distinct from non-chordate deuterostomes and protostomes (Arendt *et al*. 2001; Christiaen *et al*. 2007) and has led to the suggestion that a ‘new mouth’ is a chordate innovation (Christiaen *et al*. 2007; Hejnol & Martindale 2008).

In addition to its role in chordate oral ectoderm specification, *Pitx* involvement in left–right asymmetry downstream of *Nodal* signalling is conserved across deuterostomes (Duboc & Lepage 2008) and probably Bilateria (Grande & Patel 2009). It has thus been hypothesized that the evolution of the ‘new’ chordate mouth was the result of the evolution of a stomodeal *Pitx* expression domain that was under the control of *BMP* signalling and distinct from the asymmetric domain of *Pitx* expression that was downstream of *Nodal* (Christiaen *et al*. 2007). Evidence for the distinct regulation of these domains of expression has been uncovered in both mouse (Goodyer *et al*. 2003; Shiratori *et al*. 2006) and *Ciona* (Christiaen *et al*. 2005).

Against this background, the correspondence between the expression of *AmphiMsxlx*, *AmphiNedxa*, *AmphiNedxb* and *AmphiNKx7* and the subsequent asymmetric development of the pharyngeal apparatus in amphioxus is striking, and the expression is likely, from its spatial and temporal profile, to be downstream of the conserved *Nodal*–*Pitx* symmetry-breaking pathway (Yasui *et al*. 2000; Boorman & Shimeld 2002; Yu *et al*. 2002). We propose that the morphogenetic asymmetry of the amphioxus embryo/larva is dependent upon these ancient homeobox genes that are integrated downstream of the ancestral symmetry-breaking pathway. Under this hypothesis, amphioxus would represent an ancestral developmental gene-regulatory programme, where *Pitx* is expressed asymmetrically and is responsible for directing subsequent asymmetric pharyngeal (including oral and pre-oral) development, with *Msxlx*, *Nedx* and *NKx7* being part of the ancestral, asymmetric pharyngeal patterning system.

**Figure 5.**
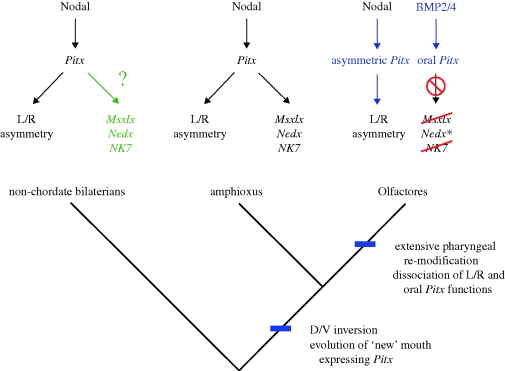
A hypothesis for the evolution of pharynx development. The evolution of chordates was marked by the relocation of the mouth to a *Pitx*-expressing territory, the expression of which may have been controlled by BMP signalling (Olfactores) or Nodal signalling (amphioxus). In the Olfactores lineage, the pharynx has undergone significant modification with the oral function of *Pitx* being controlled independently of the asymmetry function (blue). *Msxlx*, *Nedx* and *NK7*, which in amphioxus are putatively downstream of *Pitx* have been lost from Olfactores (red), except two Nedx paralogues in the appendicularian *Oikopleura* (*). The expression of *Msxlx*, *Nedx* and *NK7* in non-chordate bilaterians is unexplored (green).

In Olfactores (vertebrates+urochordates), the stomodeal function of *Pitx* (which is under the control of BMP signalling) has dissociated from the asymmetric function (under the control of Nodal signalling), and this correlates with the loss of asymmetry in pharyngeal development (figure 5). The loss of the genes examined here from vertebrates, would thus be correlated with a loss of ancestral pharyngeal developmental programmes that paved the way for the evolution of the neural crest-dependent pharyngeal development characteristic of vertebrates (Gans & Northcutt 1983; Graham 2001; Ota *et al*. 2007). In light of this suggestion, the expression of the *Oikopleura dioica Nedx* paralogues, which represent the only one of the ANTP-class homeobox families discussed here that is retained in a member of Olfactores, will be of considerable interest. A direct prediction of the model presented here would be that the *Oikopleura* genes are expressed in a novel and species-specific fashion that is distinct from BMP-dependent oral *Pitx* expression.

Of course, it remains possible that amphioxus pharyngeal development is derived relative to the chordate ancestor, with the asymmetric role of the genes examined here representing a co-option of ancient genes for a novel function. To resolve this uncertainty, comparative developmental data from non-chordate phyla will be necessary, especially from enteropneusts, which pattern their anteroposterior and dorsoventral axes in a comparable way to chordates (Lowe *et al*. 2003, 2006) and represent a crucial reference for reconstructing the ancestral deuterostome (Gerhart 2006), assuming that the genes discussed here are present in enteropneust genomes. Interestingly, an expression domain of *Pitx* in the model hemichordate *Saccoglossus kowalevskii* (Lowe *et al*. 2006) is reminiscent of the position of the chordate mouth in the conserved ectodermal expression map, and corresponds to the site where the proboscis pore later forms, the structure that indicates left–right asymmetry in this animal (Lowe *et al*. 2003).

## 5. Conclusion

We have described the expression of four genes from three ancient ANTP-class homeobox families in the basal chordate lineage of amphioxus. These three ancient gene families were all lost during vertebrate evolution. It is striking that the expression of these ‘lost’ genes is both transient and spatially restricted, and the structures that the genes are expressed in in amphioxus have been extensively modified during chordate evolution, namely the pharynx and the anterior CNS. This raises the distinct possibility that these genes have been ‘sidetracked’ into restricted developmental roles that were dispensed with during vertebrate evolution. The role of gene loss in the evolution of development has not been widely considered (reviewed in De Robertis 2008), but loss of ancient genes is clearly widespread (e.g. Kortschak *et al*. 2003; Wyder *et al*. 2007; Kuraku *et al*. 2008; Takahashi *et al*. 2009). A more complete appreciation of the role of gene loss in evolutionary developmental biology will come from extending the type of comparative genomic and embryological work highlighted here.

Irrespective of the ancestral role of *Msxlx*, *Nedx* and *NKx7*, it is clear that the evolution of the vertebrates was accompanied not just by the addition of developmental networks and structures (like the telencephalon and neural crest) onto a pre-existing amphioxus-like body plan, but that some ancestral gene-regulatory networks were dismantled leading ultimately to the loss of some of the constituent genes.
